# Dental Department

**Published:** 1900-01

**Authors:** F. S. Casper

**Affiliations:** Austin, Texas


### Dental Department.

F. S. CASPER, D. D. S., AUSTIN, TEXAS.

Dentistry Retrograding.—It seems to be the desire of a great many dentists in Texas to compete for public patronage, in price, rather than for skill and excellence. This is a step in the wrong direction, and will eventually, if persisted in, lower the standard of our profession as sure as the sun shines. Let us consider for a moment the matter from a mechanical standpoint. Ask any intelligent dentist if in case he were compelled to wear a set of artificial teeth, what kind of a set, and on what material he would want them made? And we will venture the assertion that in the majority of cases the answer would be, "continuous gum" or "gold." If any one will take the trouble to examine a mouth wherein a set of teeth has been worn, either of vulcanized rubber or celluloid, he will find the membrane congested, soft and spongy to the touch, and with irregular, white spots or patches, especially in the region of the ruga. He will also find the surface of the plate coated with a film or paste of food, which is anything else than suggestive of a sanitary condition. Now, this condition does not occur with metal plates—neither gold, platinum or silver, or the gold-lined tops of rubber plates. If this condition of things were explained to the patients they would soon elect to have none other than the best.

The mouth, of all the organs of the human system, should receive the most careful consideration as to its sanitation. What person will you find who would wear soiled unsanitary garments over his body? Then why would one place an artificial appliance in his mouth, the gateway to health, if he knew that is so doing he was placing there, simply, an incubator to breed germs of disease.

Instruct your patients. Take the trouble to enlighten them upon this all important question, and my word for it, you will find, that instead of their shopping for the lowest bidder, they will reverse their tactics, and look for the most skillful surgeon dentist.

---

ARE DENTISTS DOCTORS?—The called session of the Twenty-fifth Legislature, which convened at Austin, Texas, May 22nd, 1897, passed a law taxing physicians; an occupation tax of five dollars, which, of course, means ten dollars, as the county and city or town in which one practices adds on five more. The law reads as follows:

"An Act to amend Article 5049 of the Revised Statutes, relating to general occupation taxes.

"Sub. 13. From every physician, surgeon, oculist or other specialist of any kind, traveling from place to place in the practice of his profession, an annual tax of fifty dollars; from every dentist five dollars.

"Sub. 14. From every local practicing physician, surgeon, veterinary surgeon, or any medical or surgical specialist, an annual tax of five dollars."

We have heard that when an old experienced "bee" hunter wishes to locate a swarm of wild bees, he goes out among the wild flowers in the fields and watches the direction of the bees when they leave the flowers, and as soon as that detail is settled, he sprinkles a little sulphur on the wings of one or two of the little insects, and when they reach camp there is consternation in the hive, and by the buzzing the hunter learns where the bee lives.

We do not claim that the Legislature sprinkled any sulphur upon the physicians of Texas, but that "occupation tax" certainly set them to working like bees; they resolved and resoluted, pawed the ground and hooked the bushes, and the consequence was as follows:

"OCCUPATION TAX REPEALED.—PHYSICIANS.

"S. S. B. No. 19. Chapter CXL.

"An Act to repeal Subdivision 14, of Article 5049, Chapter 18, Title 104, of the Acts of the First Called Session of the Twenty-fifth Legislature, relating to occupation tax on physicians and surgeons.

"Be it enacted by the Legislature of the State of Texas:

"SECTION 1. That Subdivision 14, of Article 5049, Chapter 18,

Title 104, of the 'Acts of the First Called Session of the Twenty-fifth Legislature' be, and the same is, hereby repealed.

"SEC. 2. Whereas, the *collection* of occupation taxes on physicians *and* surgeons is *unjust*, creates an imperative *public* necessity that the constitutional rule requiring bills to be read on three several days be suspended, and that this act take effect from and after its passage. Approved June 5, 1899."

Notwithstanding the imperative necessity above alluded to, we see from the Galveston *News* that the tax commission, presided over by the Governor, have decided to make the physician's tax twenty-five dollars, and the dentist five dollars.

The dentist has been compelled to pay this unjust tax for a long time. We say unjust,—for in his case it is clearly a discrimination. Dentistry evidently means something. Many look at the calling of dentistry as purely mechanical, and if the law makers take the same view of dentistry (?) then in justice to the trade (?) we should not be taxed; other mechanics are not taxed. The schools in which a dentist graduates are called colleges of "Dental Surgery" or "Oral Surgery," His degree is "Doctor of Dental Surgery." Now, if "physicians and surgeons and other specialists" are not taxed, why should a dentist be required to pay this "unjust" tax?

NOTE.—Since writing the above we notice that in the House of Representatives (Congress), December 5, 1899, Congressman Otey introduced a bill, which was referred to the Committee on Military Affairs and ordered to be printed, "to provide for the appointment of *dental surgeons* for service in the United States army."

[Graduates of dental colleges are recognized as belonging to the medical profession; dentistry is a branch of medicine, and there is a Section on Dentistry in the American Medical Association.—DANIEL.]

---

THE TEETH OF RACES.—Some time in the year 1877 or 1878, the question of "The Teeth of Races" occupied a prominent place in all the dental journals published in the United States, and many able writers handled the subject, until the field seemed entirely covered. There were some who claimed that the teeth of the negro race, on account of the coloring matter contained in their blood, were stronger and more dense than those of the whites, and cited many instances, or references to prove their claim. In the early future we will give our humble opinion deduced not from the fact that we ever practiced among the Royal Families of Africa, but that we did practice for a number of years in Mississippi where the negroes out number the whites, eight to one; and although we did not practice for the Sons of Ham, we were often called upon to extract teeth for them and in so doing, the fact that we knew his previous condition of servitude, enabled us to form a definite opinion in regard to the strength of his teeth and bones generally.

In this connection, and by way of casting a "shadow before coming events," we here produce on able and interesting article published some years ago in the *Independent Practitioner:*

*"Palazzo Marotti, 172 Via Nazionale, Rome, Italy.*

"I was very much pleased with your article in the October number upon 'Conditions of the Teeth of Certain Pre-historic American Races.' I am especially glad, because I am working on the same line myself, in examining into the teeth of the Etruscan people, a race which was old when the ancient Romans began their wonderful career.

"Last May, I was requested by Prof. Helbig, of the German Archæological Society of Rome, to make a report on my investigations at Corneto Tarquirius, which I hope to have ready by January next. I expect to have good drawings made of two partial sets of teeth inserted on gold, which were taken from an Egyptian tomb, dating back five hundred years before the Christian era. I found evidences of nearly all the dental lesions which now torment the human race.

"For several years I have been skeptical on the theories, certainties, and infallible proofs of our professional authorities, who were feeling their way in the twilight of doubtful evidence, and, therefore, I decided, if possible, to 'go back of the returns' and see for myself, little dreaming that anyone on your side of old ocean was doing likewise.

"I am satisfied that your conclusions are correct, and that dental diseases are as old as the world, and not a modern new departure. As I am yet in 'the woods' of investigation, I will not crow, but I feel that no amalgam 'new departure' can compare in results with the departure you have made.

"In the meanwhile, believe me,

"Sincerely yours,

"J. G. Van Marter, A. B., D. D. S."

We have never been so fortunate as to see the final report of this investigator, but have seen reports from others who were similarly engaged, and we incline to the opinion that the coloring matter in the blood has very little, indeed, if anything, to do with the density of the structure of the teeth. We do not wish to be understood as saying that the color of the teeth does not indicate density; of this fact, there is no question.

Doctor Edwin T. Darby, of Philadelphia, in the same issue of the *Practitioner* says:

"I have read with much interest your communication on 'The Condition of the Teeth of Certain Pre-historic Races,' and your conclusions are so at variance with my former belief that it makes me wonder why the teeth of the pre-historic man of North America should show so much disease, while those of the ancient Egyptian indicate so little."

Speaking further of his investigations of the mummies in the pits at Sakkarah, he says: "With the aid of two Egyptian guides I pulled them out, unwound the linen bandages from the head and examined the teeth. I can not remember how many I took out—but dozens of them at least—and examined hundred of others which had already been taken out and unwound. Instead of finding caries of the teeth, contracted jaws, or irregularities, I found splendid teeth, large, well-formed jaws, and, with one or two trifling exceptions, no irregularity in the position of the teeth in the arch.

"I came away from the East with the opinion pretty well fixed in my mind that the earlier inhabitants of that part of the world had little or no need of dentists."

